# Mental health literacy in patients with acute myocardial infarction: a cross-sectional registry-based study

**DOI:** 10.3389/fpsyt.2024.1444381

**Published:** 2024-11-13

**Authors:** Inge Kirchberger, Simone Fischer, Philip Raake, Jakob Linseisen, Christine Meisinger, Timo Schmitz

**Affiliations:** ^1^ Epidemiology, Faculty of Medicine, University of Augsburg, Augsburg, Germany; ^2^ Department of Cardiology, Respiratory Medicine and Intensive Care, University Hospital Augsburg, Augsburg, Germany; ^3^ Institute for Medical Information Processing, Biometry and Epidemiology - IBE, Ludwig-Maximilians Universität (LMU) Munich, Munich, Germany

**Keywords:** mental health literacy, myocardial infarction, depression feldfunktion geändert, help seeking behavior, education

## Abstract

**Introduction:**

This study aimed to explore mental health literacy (MHL) and its related factors in a cross-sectional, registry-based sample of patients after acute myocardial infarction (AMI).

**Methods:**

All survivors of AMI between 2017 and 2019 from the Myocardial Infarction Registry Augsburg (n=1.712) received a postal questionnaire on MHL (Mental Health Literacy Scale (MHLS-GER)) and single questions on experiences with and information on mental disorders in 2023. The response rate was 49.9%. Logistic and linear regression models were used to investigate the associations between these variables and sociodemographic factors.

**Results:**

In the sample of 855 patients (77.5% male, mean age 71.4 ± 10.9 years), 30.0% had experienced mental problems about 5 years after AMI. Among these, 17.4% received psychotherapy and 26.1% psychotropic drugs. Information about possible mental problems after their AMI was obtained from a physician by 30.8% of the patients and in a rehabilitation setting by 46.4%, respectively. Of the patients, 26.2% wished to receive more information on mental problems after AMI. MHLS-GER subscale scores ranged between and 54 (“Social distance”) and 76 (“Information seeking”) (best score 100). Age was the most important factor that was significantly associated with the report of mental health problems, a perceived lack of information, help seeking behavior and treatment, and MHL.

**Discussion:**

Elderly and poorly educated patients were at risk of poor MHL. Further studies are required to specify the role of MHL in post-AMI life and health care.

## 1 Introduction

The concept of mental health literacy (MHL) was first introduced in 1997 by Jorm et al. who described it as “knowledge and beliefs about mental disorders which aid their recognition, management or prevention” (1). More current definitions include a number of additional components such as understanding how to obtain and maintain good mental health and prevent mental disorders, recognition of when a disorder is developing, knowledge of help-seeking options and treatments available, knowledge of effective self-help strategies for milder problems and first aid skills to support others who are developing a mental disorder or are in a mental health crisis (2).

Acute myocardial infarction (AMI) is one of the leading causes of mortality worldwide (3) with a declining short-term mortality in industrialized countries due to advances in treatment and prevention procedures (4). Hence, in ageing societies the lifespan after an AMI is extending and a closer examination of factors affecting post-AMI quality of life and clinical outcomes of AMI survivors is needed. Mental disorders are major influencing factors and there is convincing scientific evidence of a strong association between AMI and depression and anxiety disorders (5–9). Recent meta-analyses consistently reported a pooled prevalence of depression of about 28% among persons with AMI (8, 9). The pooled prevalence of moderate to severe anxiety symptoms was 38.08% in another recent meta-analysis (10). Compared with the rates of depression and anxiety symptoms in the general population, the rates in people post-AMI are considerably higher (11–13). For instance, in the German population aged 60 to 69 years, the prevalence of depressive symptoms (PHQ-9 > 9) was 7.2% (11). Depression and anxiety were shown to have a considerable negative effect on a variety of outcomes in AMI survivors including health-related quality of life (14, 15), work-resumption (16), recurrent AMI events, and mortality (17–20). Also, a number of factors were shown to contribute to post-AMI mental health problems, such as history of depression, stressors, e.g. financial strain, poor self-rated health, low socioeconomic status, and type-D personality (21–23).

**Figure 1:**
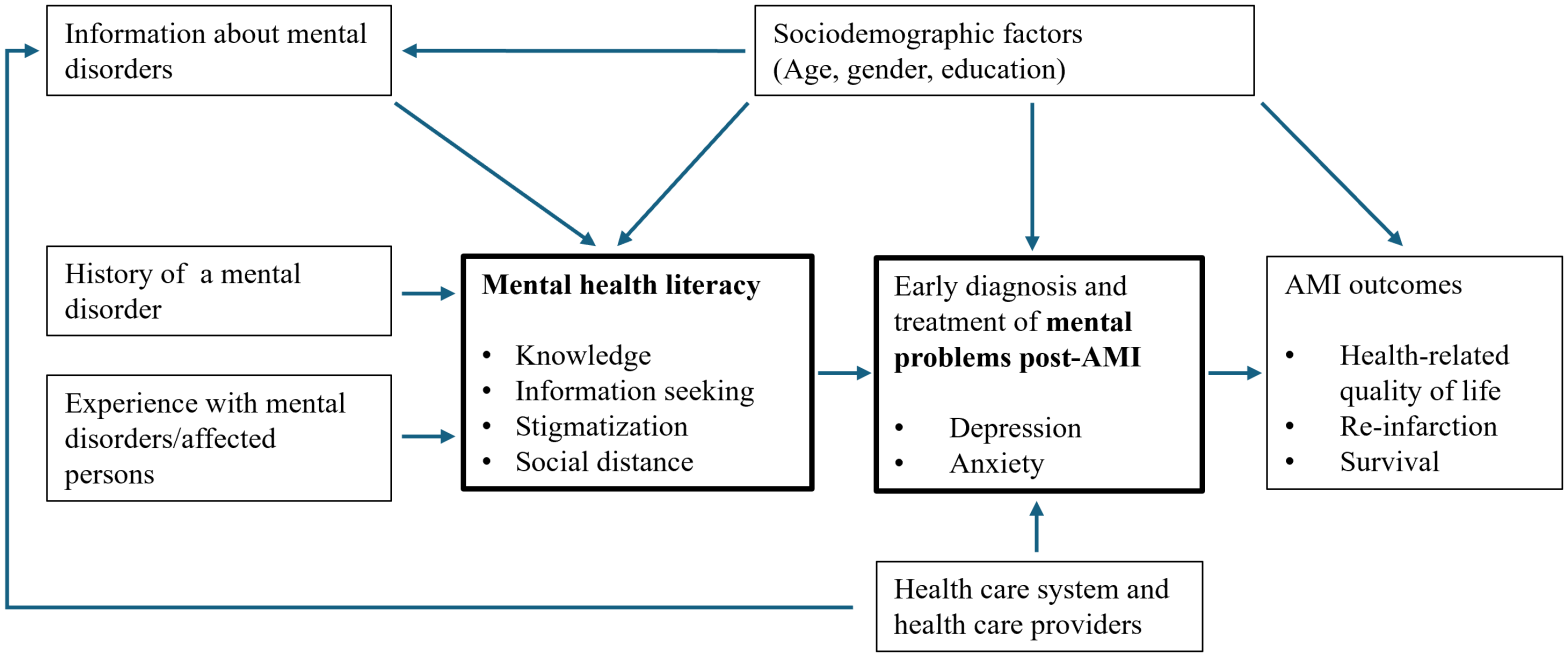
Conceptual model of the associations with mental health literacy in patients with acute myocardial infarction.

Despite the high prevalence of depression and anxiety in AMI patients and the availability of effective treatment approaches, many of these patients remain undiagnosed and untreated (24–26). A meta-analysis of 15 cohort studies in patients with AMI showed that of 2381 depressed individuals 14% used antidepressant medication (27). Compared with depression treatment rates around 30% in the general population, undertreatment seems to be more common in post AMI patients (28, 29). The specific reasons for the persisting underdiagnosis and undertreatment of mental health problems in AMI patients remain unclear (30). Besides barriers located in the health care system and – providers, and sociodemographic factors, e.g. age or education, a lack of MHL among patients with AMI may also contribute substantially to these findings (see **Figure 1**).

**Figure 1** shows the conceptual framework of the present study which proposes an effect of MHL on the timely diagnosis and treatment of mental health problems in patients with AMI which may subsequently improve AMI outcomes such as health-related quality of life, re-infarction and survival. The present study will focus on factors being associated with MHL such as sociodemographic factors, history of a mental disease, experience with mental disorders or affected persons, and provision of information on mental disorders and will explore MHL in a cross-sectional, registry-based sample of patients after AMI.

## 2 Methods

### 2.1 Design and study population

The present study used data from a postal follow-up survey on participants of the Augsburg Myocardial Infarction Registry, which was established as a part of the MONICA-project (Monitoring Trends and Determinants in Cardiovascular disease) in 1984. The study area covers the city of Augsburg, Germany, and the two adjacent counties, including a total of approximately 680,000 inhabitants. The registry continuously registers all cases of coronary death and non-fatal AMI of the study population older than 24 years. Methods of case finding, diagnostic classification of AMI as well as data quality control were detailed elsewhere (31).

The registry was approved by the ethics committee of the Bavarian Medical Association (Bayerische Landesärztekammer) and the study was performed in accordance with the Declaration of Helsinki. Written informed consent was obtained from all participants.

In April 2023, all survivors with incident or recurrent AMI admitted between 2017 and 2019 (n = 1.712) were sent a questionnaire via post and were asked to complete questions on diabetes, HRQOL, depression, fatigue, and mental health literacy. A total of 855 (49.9%) patients returned the questionnaire. From the 857 non-responders, 67 patients had died, 104 had moved with unknown address, and 42 indicated that they are not willing or able to answer the questions. The remaining 644 persons received a postal reminder, but they did not respond. No significant differences between responders and non-responders in terms of age and gender were found.

### 2.2 Survey data

Self-developed single items requested information on previous diagnosis of mental health conditions, experiences with mental disorders in the private environment or due to a professional activity, information on AMI-related mental problems by an attending physician or during a rehabilitation program, satisfaction with the amount of information and willingness to use digital information tools (see **Supplementary Table 1**). The participants were also asked whether they had any mental problems after the AMI and, if so, which problems they had, whether they sought help from there private environment or from professionals, and whether they received psychotherapy or pharmacotherapy.

MHL was assessed using the German version of the ‘Mental Health Literacy Scale’ (MHLS) (32). The 35 items of the MHLS address the ability to recognize specific mental disorders, the attitudes that promote recognition and appropriate help-seeking, as well as the knowledge of how to seek mental health information, of risk factors and causes, of self-treatments, and of professional help available. The MHLS has demonstrated sufficient reliability and validity in different languages (33–38). The psychometric evaluation of the German version of the MHLS (MHLS-GER) in different samples of the German population suggests the scoring of four subscales, namely “Knowledge” (11 items), “Information seeking” (4 items), “Stigmatization” (9 items), and “Social distance” (7 items) (39). Since 4- and 5-point Likert scales were used as response scales for the different subscales, all subscale scores were transformed to a 0 to 100-point scale [(sum of item scores - minimum sum score)/(maximum sum score – minimum sum score) * 100] with higher scores indicating better MHL.

Depressive symptoms were assessed with the depression module of the Patient Health Questionnaire (PHQ) (40–42). The PHQ-9 consists of 9 items with response options from 0 to 3 (never to nearly every day), resulting in a score ranging from 0 to 27. A score less than five can be interpreted as the absence of depressiveness. Values between five and ten constitute a mild degree of depressiveness. Values of ten and higher can be subdivided into moderate (ten to 14), moderately severe (15 to 19), and severe (20 to 27) depressiveness (27). The German version of the PHQ-9 showed good psychometric properties (43).

### 2.3 Data analysis

Continuous variables were described as means ± standard deviations (SD) and categorical variables as absolute and relative frequencies. Subgroup differences in MHLS-GER scores were tested using Mann-Whitney U-Test or Kruskal-Wallis test. Logistic regression models were performed to estimate the relation between binary outcome variables and the independent variables age, gender, and educational attainment. Multivariable linear regression models were performed to investigate the associations between the four subscales of the MHLS-GER (dependent variables) and sociodemographic or health-related variables (independent variables). The independent variables were selected based on results from available publications. The assumptions of multivariable linear regression were ensured. Effect size was determined using Cohen’s F**^2^.** For statistical tests an alpha level of 0.05 was defined. Due to the explorative study approach no adjustment for multiple testing was applied. Statistical analyses were performed using SAS Version 9.4.

## 3 Results

### 3.1 Sample characteristics

The demographic and clinical characteristics of the participants are shown in **Table 1**. The mean age of the enrolled participants at the time of the follow-up survey was 71.4 ± 10.9 years (Range 38 to 95 years) with 77.5% men (mean age 70.9 ± 10.8 years) and 22.5% women (mean age 73.3 ± 11.0 years). The mean time between AMI and survey was 4.8 ± 0.9 years.

**Table 1:** Sample characteristics.

	*n*	*%*
Male sex	663	77.5
Female sex	192	22.5
School education > 9 years	406	48.1
Currently employed	213	25.0
Marital status		
Married	608	71.5
Single	59	6.9
Divorced	76	8.9
Widowed	108	12.7
Comorbid conditions		
Hypertension	625	73.1
Dyslipidemia	494	57.8
Diabetes	209	25.1
Obesity (>30 kg/m²)	214	25.6
Prior infarction	113	13.2
Smoking status		
Smoker	210	24.7
Ex-Smoker	306	36.0
Never smoker	335	39.3
Type of infarction		
STEMI	327	38.3
NSTEMI	398	46.5
Bundle branch block	75	8.8
Not defined	55	6.4
Treatment		
PTCA	729	85.5
Aortocoronary bypass	81	9.6
Depression^1^		
No	450	53.3
Mild	279	33.1
Moderate	83	9.8
Moderately severe	24	9.8
Severe	8	1.0

AMI, acute myocardial infarction; STEMI, ST-segment elevation myocardial infarction; NSTEMI, Non ST-segment elevation myocardial infarction; PTCA, percutaneous transluminal coronary angioplasty; ^1^ Patient Health Questionnaire (PHQ-9).

### 3.2 Experiences with mental disorders and information about mental problems after AMI

**Figure 2:**
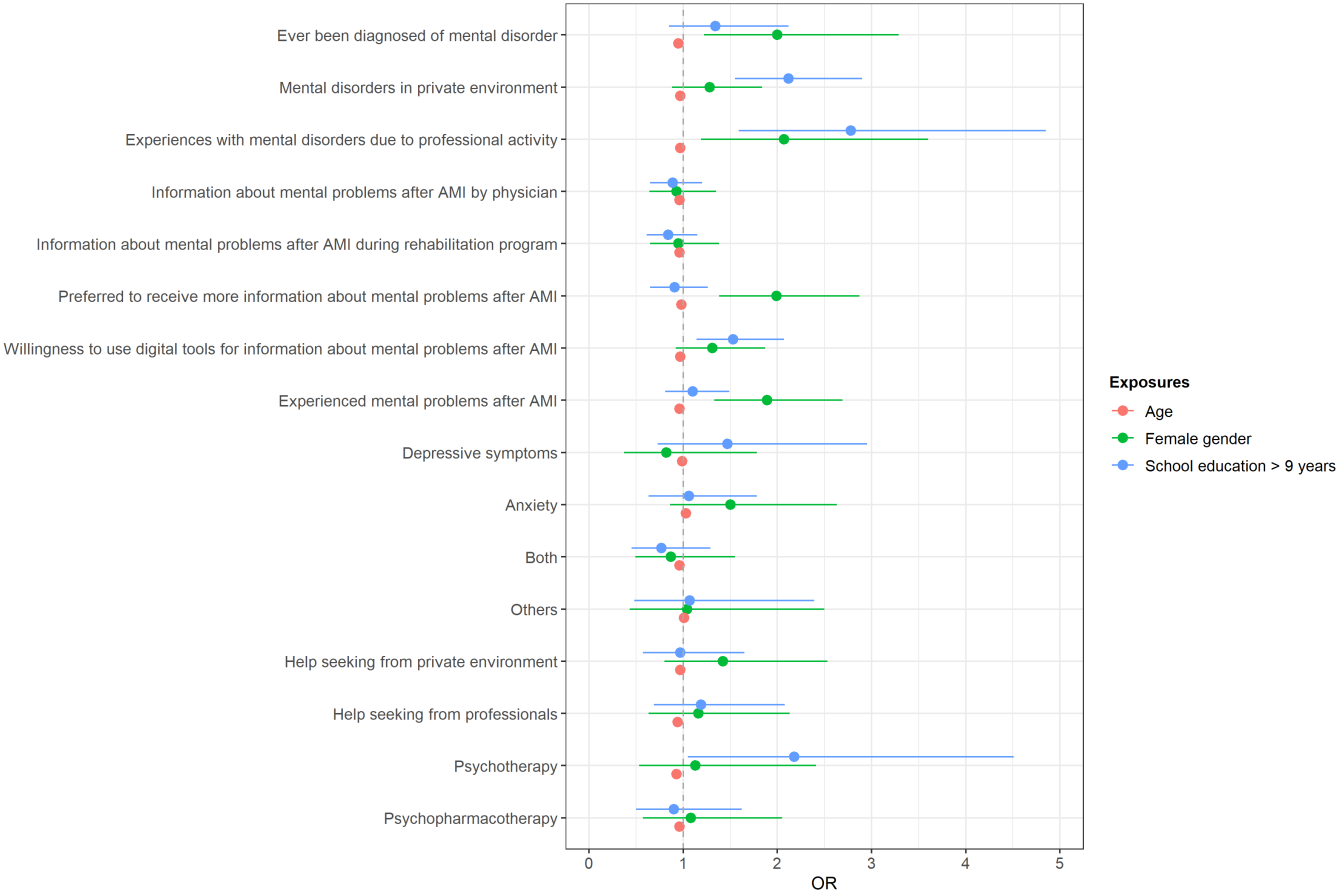
Multivariable logistic regression models. Outcomes: Experiences with mental disorders and information about mental problems after acute myocardial infarction, exposers: age, gender and education (OR, Odds ratio).

The results stratified by gender, age and education are shown in **Tables 2**, **3**. In brief, 10.6% of the study participants have ever been diagnosed of a mental disorder. Information about possible mental problems after their AMI were provided by a physician for 30.8% of the patients and during rehabilitation information was provided for 46.4% of the patients who received in-hospital or out-hospital rehabilitation (80.4%) A perceived lack of information was reported by 26.2% of the patients. About 31% of the patients would be willing to use digital tools for being informed about mental health problems after AMI. Mental problems after AMI were reported by 30.0% of the study participants, with 34.3% who sought help from the private environment and 32.6% from health professionals. Of those with mental problems after AMI, 17.4% received psychotherapy and 26.1% were treated with psychotropic drugs. Age emerged as the most important independent factor that was significantly associated with the responses (see **Figure 2**; **Supplementary Table 2**). Younger aged patients were significantly more likely to have a history of mental disorder, to experience mental problems after AMI, and to receive information about possible mental problems after AMI on the one hand, but to perceive an information lack on the other hand. Furthermore, younger patients were more likely to be willing to use digital tools to obtain more information, to seek help from the private environment and from health professionals and to be treated with psychotherapy or psychotropic drugs. Higher level of school education was significantly associated with mental disorders in the private environment, experiences with mental disorders due to professional activity, willingness to use digital tools for information about mental problems after AMI, and the use of psychotherapy. Women were significantly more likely than men to have a prior diagnosis of a mental disorder, to experience mental problems after AMI, to have experiences with mental disorders due to professional activity, and to prefer getting more information about mental problems after AMI.

**Table 2:** Experiences with mental disorders and information about mental problems after acute myocardial infarction (AMI) stratified by gender, and education.

	Total		Gender				Education (years)			
	n=855		Male (n=663)		Female (n=192)		≤ 9 (n=438)		> 9 (n=406)	
	*n*	*%*	*n*	*%*	*n*	*%*	*n*	*%*	*n*	*%*
Ever been diagnosed of mental disorder	89	10.6	64	9.8	30	15.9	38	8.8	50	12.4
Mental disorders in private environment	239	28.3	181	27.7	58	30.2	91	20.9	147	36.8
Experiences with mental disorders due to professional activity	66	7.9	44	6.7	22	11.7	19	4.4	47	11.8
Information about mental problems after AMI by physician	260	30.8	206	31.5	54	28.4	134	30.9	120	30.1
Information about mental problems after AMI during rehabilitation program	319	46.4	254	47.2	65	43.6	163	47.4	151	45.2
Preferred to receive more information about mental problems after AMI	220	26.2	150	23.0	70	37.2	112	25.9	105	26.5
Willingness to use digital tools for information about mental problems after AMI	267	31.9	202	31.0	65	35.1	117	27.2	148	37.4
Experienced mental problems after AMI	255	30.0	182	27.7	73	38.2	125	28.7	129	32.0
Depressive symptoms	40	4.7	30	4.5	10	5.2	16	3.7	23	5.7
Anxiety	97	11.4	64	9.7	33	17.2	48	11.0	49	12.1
Both	101	11.9	74	11.2	27	14.1	52	11.9	49	12.1
Others	27	3.2	19	2.9	8	4.2	13	3.0	14	3.5
Help seeking from private environment	86	34.3	58	32.2	28	39.4	41	33.3	44	34.7
Help seeking from professionals	83	32.6	58	31.9	25	34.3	36	28.8	46	35.7
Psychotherapy	44	17.4	30	16.7	1	19.2	14	11.2	30	23.6
Psychopharmacotherapy	66	26.1	4	26.0	19	26.4	32	25.6	33	26.0

**Table 3:** Experiences with mental disorders and information about mental problems after acute myocardial infarction (AMI) stratified by age.

	Age (years)							
	≤ 60 (n=156)		61- 70 (n=240)		71-80 (n=236)		> 80 (n=223)	
	*n*	*%*	*n*	*%*	*n*	*%*	*n*	*%*
Ever been diagnosed of mental disorder	29	18.7	30	12.7	20	8.6	10	4.6
Mental disorders in private environment	64	41.0	72	30.5	64	27.6	39	17.6
Experiences with mental disorders due to professional activity	14	9.0	26	11.0	20	8.7	6	2.8
Information about mental problems after AMI by physician	66	42.6	92	39.0	62	26.7	40	18.1
Information about mental problems after AMI during rehabilitation program	89	63.1	111	54.4	59	33.0	60	36.8
Preferred to receive more information about mental problems after AMI	50	32.5	66	27.9	54	23.5	50	22.7
Willingness to use digital tools for information about mental problems after AMI	68	44.4	74	31.5	72	31.3	53	24.2
Experienced mental problems after AMI	76	48.7	78	32.8	51	21.8	50	22.6
Depressive symptoms	12	7.7	16	6.7	7	3.0	5	2.2
Anxiety	22	14.1	27	11.3	23	9.8	25	11.2
Both	39	25.0	33	13.8	16	6.8	13	5.8
Others	8	5.1	6	2.5	6	2.5	7	3.1
Help seeking from private environment	31	40.8	27	34.6	16	32.0	12	25.5
Help seeking from professionals	36	47.4	28	35.9	12	23.5	7	14.0
Psychotherapy	2	32.9	12	15.6	4	7.8	3	6.1
Psychopharmacotherapy	28	36.8	19	25.0	9	17.7	10	20.0

### 3.3 Factors associated with MHL

From the 855 respondents, 139 (16.3%) had missing scores in at least one MHLS-GER subscale. The subscale “Knowledge” was not completed by 11.5% of the participants. Participants with missing scores in at least one MHLS-GER subscale significantly differed from those who completed the questionnaire. They were significantly older (mean age 74.9 vs. 70.8 years, p<0.0001), had less often experienced mental problems after AMI (20.7% vs. 31.8%, p=0.0102), and had less experiences with mental disorders in the private environment (18.7% vs. 30.0%, p=0.0072).

Mean scores for the MHLS-GER subscales in the total sample were 64.03 ± 19.61 for “Knowledge”, 76.35 ± 22.37 for “Information seeking”, 74.17 ± 17.83 for “Stigmatization”, and 54.16 ± 21.91 for “Social distance”. Significantly higher MHLS-GER scores (indicating better MHL) in all four subscales were found in younger patients compared with older ones, and in patients who were educated about mental problems after AMI during a rehabilitation program compared with those who received no information (see **Supplementary Table 3**).

Furthermore, school education, prior diagnosis of a mental disorder, experiences with mental disorders in private environment, information about mental problems after AMI by a physician, perceived lack of information about mental problems after AMI, and PHQ-9 scores were significantly associated with three out of four MHLS-GER subscale scores. Experiences with mental disorders due to professional activity were significantly associated with better scores in two MHLS-GER subscale scores.

The multivariable linear regression models confirmed most of the univariable associations (see **Tables 4**–**7**). Younger age was significantly associated with better MHL in all four subscales. Higher school education, information about mental problems after AMI during a rehabilitation program, and experiences with mental disorders in private environment were significantly associated with better scores in three subscales. Patients with a prior diagnosis of a mental disorder and patients with lower PHQ-9 score had significantly better scores in two subscales compared with their counterparts. Information about mental problems after AMI by a physician was significantly associated with better scores in the subscale “Social distance”.

**Table 4:** Multivariable linear regression model of the MHLS-GER subscale “Knowledge”.

	Knowledge (n=587)				
	*ß*	95% CI		*p*	*F^2^*
		*LL*	*UL*		
Female gender	3.71	0.08	7.33	**0.0450**	0.0070
Age	-0.19	-0.33	0.05	**0.0095**	0.0118
School education > 9 years	7.90	4.88	10.93	**<.0001**	0.0428
Prior mental disorder	4.22	-0.87	9.30	0.1039	0.0040
Mental disorders in private environment	6.50	3.17	9.83	**0.0001**	0.0261
Experiences with mental disorders due to professional activity	2.62	-2.55	7.80	0.3193	0.0138
Information about mental problems after AMI by physician	-2.38	-6.34	1.58	0.2377	0.0016
Information about mental problems after AMI during rehabilitation program	5.89	2.10	9.67	**0.0023**	0.0092
PHQ-9 score	-0.10	-0.47	0.28	0.6132	0.0020

Significant results (p < 0.05) are highlighted in bold type.

CI, confidence interval; LL, lower limit; UL, upper limit; AMI, acute myocardial infarction; PHQ-9, Patient Health Questionnaire.

**Table 5:** Multivariable linear regression model of the MHLS-GER subscale “Information seeking”.

	Information seeking (n=607)				
	*ß*	95% CI		*p*	*F^2^*
		*LL*	*UL*		
Female gender	0.90	-3.32	5.11	0.6764	<.0001
Age	-0.33	-0.50	0.17	**<.0001**	0.0273
School education > 9 years	4.11	0.65	7.58	**0.0199**	0.0076
Prior mental disorder	0.08	-5.70	5.86	0.9785	-0.0014
Mental disorders in private environment	-0.45	-4.28	3.38	0.8173	<.0001
Experiences with mental disorders due to professional activity	2.59	-3.38	8.56	0.3944	<.0001
Information about mental problems after AMI by physician	-0.39	-4.94	4.17	0.8682	<.0001
Information about mental problems after AMI during rehabilitation program	4.52	0.17	8.87	**0.0416**	0.0095
PHQ-9 score	-1.14	-1.57	-0.71	**<.0001**	0.0450

Significant results (p < 0.05) are highlighted in bold type.

CI, confidence interval; LL, lower limit; UL, upper limit; AMI, acute myocardial infarction; PHQ-9, Patient Health Questionnaire.

**Table 6:** Multivariable linear regression model of the MHLS-GER subscale “Stigmatization”.

	Stigmatization (n=608)				
	*ß*	95% CI		*p*	*F^2^*
		*LL*	*UL*		
Female gender	3.16	-0.12	6.44	0.0590	0.0060
Age	-0.22	-0.34	0.09	**0.0008**	0.0189
School education > 9 years	5.44	2.74	8.14	**<.0001**	0.0250
Prior mental disorder	6.90	2.38	11.42	**0.0028**	0.0150
Mental disorders in private environment	4.53	1.54	7.52	**0.0031**	0.0150
Experiences with mental disorders due to professional activity	4.22	-0.44	8.88	0.0762	0.0100
Information about mental problems after AMI by physician	0.60	-2.95	4.15	0.7394	<.0001
Information about mental problems after AMI during rehabilitation program	2.80	-0.59	6.20	0.1051	0.0208
PHQ-9 score	-0.87	-1.21	-0.53	**<.0001**	0.0430

Significant results (p < 0.05) are highlighted in bold type.

CI, confidence interval; LL, lower limit; UL, upper limit; AMI, acute myocardial infarction; PHQ-9, Patient Health Questionnaire.

**Table 7:** Multivariable linear regression model of the MHLS-GER subscale “Social distance”.

	Social distance (n=595)				
	*ß*	95% CI		*p*	*F^2^*
		*LL*	*UL*		
Female gender	1.86	-2.16	5.89	0.3640	0.0015
Age	-0.54	-0.69	-0.38	**<.0001**	0.0782
School education > 9 years	-0.90	-4.25	2.44	0.5951	<.0001
Prior mental disorder	7.03	1.45	12.61	**0.0136**	0.0108
Mental disorders in private environment	3.94	0.24	7.64	**0.0369**	0.0077
Experiences with mental disorders due to professional activity	2.79	-3.10	8.68	0.3532	<.0001
Information about mental problems after AMI by physician	-5.55	-9.96	-1.13	**0.0139**	0.0085
Information about mental problems after AMI during rehabilitation program	6.23	2.04	10.42	**0.0036**	0.0159
PHQ-9 score	-0.40	-0.82	0.02	0.0612	0.0116

Significant results (p < 0.05) are highlighted in bold type.

CI, confidence interval; LL, lower limit; UL, upper limit; AMI, acute myocardial infarction; PHQ-9, Patient Health Questionnaire.

## 4 Discussion

Among the factors which are supposed to be associated with MHL according to the conceptual model displayed in **Figure 1**, age was the most relevant variable associated both with MHL and with other variables expected to be associated with MHL, such as information about mental disorders. Older persons were less likely to report mental problems after AMI than younger ones. However, older persons also were less likely to receive information about AMI by a doctor or in a rehabilitation setting and they had less often personal experiences with mental disorders than younger patients. Knowledge of mental health problems and proximity to individuals with mental health problems have been identified as major aspects of MHL which may also facilitate an early diagnosis and treatment of mental disorders (44). Indeed, the older patients who reported mental problems after AMI showed reduced help seeking behavior and were less often treated with psychotherapy or pharmacological treatment compared with younger patients. These findings may be to some extent caused by the overall lower MHL reflected by poorer scores in all MHLS-GER subscales compared with younger individuals. The association of age and MHL has been only rarely investigated in older aged populations so far and showed conflicting results (45, 46). The results from the present study, however, support that older age is also associated with poorer MHL in a sample of persons with AMI aged 71 years in average.

Besides age, poor education was identified as a relevant variable shown to be significantly related with poor MHL reflected by low scores in the MHLS-GER subscales “Knowledge”, “Information seeking”, and “Stigmatization” in the present study. Comparable results were found in a number of prior studies with different age groups, nationalities and health states (46–51). Furthermore, the results from the present study confirmed other studies that found associations between MHL and gender (47, 48, 52, 53), and MHL and proximity to persons with mental disorders in the private environment (54, 55).

Information about mental health problems after AMI provided by health carers is another important factor shown to be related with MHL in the present study. More than one fourth of all study participants reported that they would have preferred to receive more information about possible mental problems after AMI. Interestingly, these patients were younger, more often women and had more often private contacts with persons with mental disorders. Those persons seem to be already sensitized for mental health issues and thus open for further information regarding their own situation.

Of interest, patients who received information about possible mental problems after AMI in the post-AMI rehabilitation setting, showed significantly better MHL than patients without being informed. This finding may point to in-hospital or out-patient rehabilitation as a meaningful setting for interventions aiming to improve MHL in patients with AMI. However, it must be considered that in Germany about 20% of the patients with AMI do not utilize rehabilitation and thus interventions on MHL should also be offered in other settings. Education and provision of information are common interventions to improve MHL and digital provision of information, e.g. by internet websites or Apps, is increasingly applied in health care (56, 57). However, the results from this study also point to possible limitations of such an approach in older-aged target groups since in the study population of AMI patients only about one third reported to be willing to receive digital information on mental health problems after AMI. Thus, it seems essential that interventions on MHL in patients with AMI consider the personal and environmental characteristics of this group and chose a suitable communication medium and setting.

The present study also demonstrated that poor MHLS-GER scores of the subscales “Information seeking” and “Stigmatization” are associated with more depressive symptomatology according to PHQ-9 scores. This finding is consistent with Ding et al. (58) who found significant independent associations between PHQ-9 scores and MHL in a sample of the Chinese population with a mean age of 70 years. Further in-depth investigations of the relationships between the different variables and MHL are needed in order to increase knowledge about mediating, moderating or causal effects (45).

The present study also points to important aspects of MHL assessment in an elderly sample of patients with a somatic disorder. First, we assume that the questions on mental health and MHL have reduced the overall response rate, which was 49% and considerably lower than in previous follow-up surveys conducted at the Myocardial Infarction Registry with response rates of e.g. 67% (59). Some comments from non-responding patients indicated that it made no sense for them to complete questions on mental health issues since they have a somatic disease. This points to the missing awareness of mental comorbidities in somatic disorders.

To our knowledge, the present study is the first which investigated MHL in patients with AMI. Strengths of this study are the large number of well-characterized consecutive AMI-patients from a population-based registry. However, we cannot exclude a selection bias towards patients with better MHL as they were more likely to complete the questionnaire. Furthermore, patients with missing scores in the MHLS-GER significantly differed from those who completed the scale. Symptoms of depression were assessed using the PHQ-9, however, a clinical diagnosis of depression was not available. Since a comparison group is missing, the MHLS-GER scores of persons with AMI cannot be compared with persons without AMI. It cannot be excluded that other relevant covariables which may be associated with MHL, e.g. nationality, migration background or others, were not considered. Furthermore, due to the cross-sectional study design, causal relationships between the variables investigated cannot be deduced. Finally, the results may not be generalizable to other age-groups, ethnic groups and non-European nationalities.

In conclusion, the present study found a considerable burden of mental health problems in patients about 5 years after their AMI. Specifically elderly and poorly educated patients were identified as a risk group of poor MHL, subsequent undertreatment and persisting impaired mental health. Further studies are required to specify the role of MHL in post-AMI life and mental health care. Based on these results, interventions aimed at improving MHL in this patient group may be developed, which should be tailored to subgroups of patients with highest risk of poor MHL such as elderly and poorly educated, should apply a suitable communication medium and setting, and should be evaluated in randomized controlled studies.

## Data Availability

The data that support the findings of this study are available from the Chair of Epidemiology, Medical Faculty, University of Augsburg but restrictions apply to the availability of these data, which are not publicly available. Data are, however, available from the authors upon reasonable request and with permission of the Chair of Epidemiology. Requests to access the datasets should be directed to Inge.Kirchberger@med.uni-augsburg.de.
