# Supplementary material for: Mental health literacy in patients with acute myocardial infarction: a cross-sectional registry-based study
Source: Front Psychiatry. 2024 Nov 13;15:1444381. doi: 10.3389/fpsyt.2024.1444381 (PMC11599234; doi:10.3389/fpsyt.2024.1444381)
Supplement: Supplementary file 1 [file Table1.docx]

Supplementary Table 1: Single questions on experience with and information on mental health problems

- Have you ever been diagnosed with a mental health condition by a physician?
- Do you have experiences with mental disorders in your close private environment ( e.g. family or friends with a mental disorder)?
- Do you have experiences with mental disorders due to your professional activity (e.g. health professional)?
- Did you receive information about the occurrence or management of mental problems (e.g. depression and anxiety) after your heart attack by your attending physician?
- Did you receive information about the occurrence or management of mental problems (e.g. depression and anxiety) after your heart attack during an in-patient or out-patient rehabilitation program?
- Would you have preferred to receive more information about possible mental problems after your myocardial infarction?
- Would you utilize digital tools in order to get more information about mental health issues after myocardial infarction?
- Did you experience mental problems, e.g. increased anxiety or depressive mood, after your myocardial infarction?
- If yes: Which mental problems did/do you experience after your myocardial infarction?
- Depressive symptoms
- Anxiety
- Both
- Other
- Did you seek help from your private environment (family, friends) because of your mental problems after myocardial infarction?
- Did you seek help from professionals (physician, psychologist, counseling center) because of your mental problems after myocardial infarction?
- Did you start a psychotherapy because of your mental problems?
- Were you prescribed drugs (e.g. antidepressants) because of your mental problems
