# Supplementary material for: Mental health literacy in patients with acute myocardial infarction: a cross-sectional registry-based study
Source: Front Psychiatry. 2024 Nov 13;15:1444381. doi: 10.3389/fpsyt.2024.1444381 (PMC11599234; doi:10.3389/fpsyt.2024.1444381)
Supplement: Supplementary file 2 [file Table2.docx]

Supplementary Table 2: Multivariable logistic regression analysis. Significant results (p < 0.05) are highlighted in bold type.

|  | Female gender | | | | Age | | | | School education > 9 years | | | |
| --- | --- | --- | --- | --- | --- | --- | --- | --- | --- | --- | --- | --- |
|  | *OR* | 95% CI | | *p* | *OR* | 95% CI | | *p* | *OR* | 95% CI | | *p* |
|  |  | *LL* | *UL* |  |  | *LL* | *UL* |  |  | *LL* | *UL* |  |
| Ever been diagnosed of mental disorder | 2.00 | 1.22 | 3.29 | **0.0063** | 0.95 | 0.93 | 0.97 | **<.0001** | 1.34 | 0.85 | 2.12 | 0.2048 |
| Mental disorders in private environment | 1.28 | 0.88 | 1.84 | 0.1938 | 0.97 | 0.95 | 0.98 | **<.0001** | 2.12 | 1.55 | 2.90 | **<.0001** |
| Experiences with mental disorders due to professional activity | 2.07 | 1.19 | 3.60 | **0.0104** | 0.97 | 0.94 | 0.99 | **0.0038** | 2.78 | 1.59 | 4.85 | **0.0003** |
| Information about mental problems after AMI by physician | 0.93 | 0.64 | 1.35 | 0.7093 | 0.96 | 0.94 | 0.97 | **<.0001** | 0.89 | 0.65 | 1.20 | 0.4289 |
| Information about mental problems after AMI during rehabilitation program | 0.95 | 0.65 | 1.38 | 0.7724 | 0.96 | 0.94 | 0.97 | **<.0001** | 0.84 | 0.61 | 1.15 | 0.2704 |
| Preferred to receive more information about mental problems after AMI | 1.99 | 1.38 | 2.87 | **0.0002** | 0.98 | 0.97 | 0.99 | **0.0367** | 0.91 | 0.65 | 1.26 | 0.5547 |
| Willingness to use digital tools for information about mental problems after AMI | 1.31 | 0.92 | 1.87 | 0.1409 | 0.97 | 0.96 | 0.98 | **<.0001** | 1.53 | 1.14 | 2.07 | **0.0050** |
| Experienced mental problems after AMI | 1.89 | 1.33 | 2.69 | **0.0004** | 0.96 | 0.94 | 0.97 | **<.0001** | 1.10 | 0.81 | 1.49 | 0.5481 |
| Depressive symptoms | 0.82 | 0.37 | 1.78 | 0.6042 | 0.99 | 0.96 | 1.03 | 0.6898 | 1.47 | 0.73 | 2.95 | 0.2789 |
| Anxiety | 1.50 | 0.86 | 2.63 | 0.1565 | 1.03 | 1.01 | 1.06 | **0.0081** | 1.06 | 0.63 | 1.78 | 0.8404 |
| Both | 0.87 | 0.49 | 1.55 | 0.6445 | 0.96 | 0.94 | 0.98 | **0.0004** | 0.77 | 0.45 | 1.29 | 0.3149 |
| Others | 1.04 | 0.43 | 2.50 | 0.9256 | 1.01 | 0.97 | 1.04 | 0.6890 | 1.07 | 0.48 | 2.39 | 0.8729 |
| Help seeking from private environment | 1.42 | 0.80 | 2.53 | 0.2342 | 0.97 | 0.95 | 0.99 | **0.0275** | 0.97 | 0.57 | 1.65 | 0.9010 |
| Help seeking from professionals | 1.16 | 0.63 | 2.13 | 0.6288 | 0.94 | 0.92 | 0.97 | **<.0001** | 1.19 | 0.69 | 2.08 | 0.5313 |
| Psychotherapy | 1.13 | 0.53 | 2.41 | 0.7470 | 0.93 | 0.90 | 0.96 | **<.0001** | 2.18 | 1.05 | 4.51 | **0.0345** |
| Psychopharmacotherapy | 1.08 | 0.57 | 2.05 | 0.8144 | 0.96 | 0.93 | 0.98 | **0.0008** | 0.90 | 0.50 | 1.62 | 0.7315 |

OR = Odds ratio; CI = confidence interval; LL = lower limit; UL = upper limit; AMI = acute myocardial infarction
