# Supplementary material for: Mental health literacy in patients with acute myocardial infarction: a cross-sectional registry-based study
Source: Front Psychiatry. 2024 Nov 13;15:1444381. doi: 10.3389/fpsyt.2024.1444381 (PMC11599234; doi:10.3389/fpsyt.2024.1444381)
Supplement: Supplementary file 3 [file Table3.docx]

Supplementary Table 3: Differences in MHLS-GER scores in subgroups of patients. Significant results (p<0.05) are highlighted in bold type.

|  | **Knowledge** | | | | **Information seeking** | | | | **Stigmatization** | | | | **Social distance** | | | |
| --- | --- | --- | --- | --- | --- | --- | --- | --- | --- | --- | --- | --- | --- | --- | --- | --- |
|  | **n** | **Mean** | **SD** | **p-value*** | **n** | **Mean** | **SD** | **p-value*** | **n** | **Mean** | **SD** | **p-value*** | **n** | **Mean** | **SD** | **p-value*** |
| Gender |  |  |  | 0.1371 |  |  |  | 0.7899 |  |  |  | **0.0296** |  |  |  | 0.1210 |
| Male | 582 | 63.43 | 19.75 |  | 613 | 76.35 | 22.57 |  | 612 | 73.55 | 17.74 |  | 596 | 53.49 | 21.83 |  |
| Female | 175 | 66.01 | 19.03 |  | 174 | 76.34 | 21.73 |  | 174 | 76.36 | 18.02 |  | 173 | 56.46 | 22.08 |  |
| Age [years] |  |  |  | **<.0001** |  |  |  | **<.0001** |  |  |  | **<.0001** |  |  |  | **<.0001** |
| ≤ 60 | 146 | 68.00 | 18.15 |  | 151 | 79.58 | 22.63 |  | 151 | 79.72 | 16.67 |  | 150 | 63.52 | 22.75 |  |
| 61- 70 | 214 | 68.04 | 18.08 |  | 224 | 79.98 | 20.10 |  | 222 | 75.94 | 16.81 |  | 210 | 57.47 | 20.52 |  |
| 71 - 80 | 211 | 62.24 | 20.84 |  | 221 | 75.31 | 21.91 |  | 220 | 74.71 | 17.92 |  | 215 | 52.11 | 20.92 |  |
| > 80 | 186 | 58.31 | 19.39 |  | 191 | 70.74 | 24.08 |  | 193 | 67.17 | 17.72 |  | 194 | 45.61 | 20.28 |  |
| Education [years] |  |  |  | **<.0001** |  |  |  | **0.0034** |  |  |  | **<.0001** |  |  |  | 0.3752 |
| ≤ 9 | 378 | 59.52 | 19.98 |  | 395 | 74.00 | 23.41 |  | 395 | 71.05 | 18.16 |  | 386 | 53.35 | 22.15 |  |
| > 9 | 370 | 69.07 | 17.54 |  | 383 | 78.67 | 21.23 |  | 382 | 77.71 | 16.68 |  | 375 | 54.96 | 21.74 |  |
| Ever been diagnosed of mental disorder |  |  |  | **0.0002** |  |  |  | 0.5939 |  |  |  | **<.0001** |  |  |  | **<.0001** |
| yes | 83 | 72.08 | 15.21 |  | 86 | 74.95 | 23.19 |  | 86 | 80.94 | 15.43 |  | 85 | 63.77 | 22.13 |  |
| no | 666 | 63.13 | 19.81 |  | 690 | 76.51 | 22.30 |  | 689 | 73.46 | 17.99 |  | 675 | 52.92 | 21.55 |  |
| Mental disorders in private environment |  |  |  | **<.0001** |  |  |  | 0.9155 |  |  |  | **<.0001** |  |  |  | **0.0002** |
| yes | 220 | 71.23 | 15.89 |  | 227 | 77.14 | 20.15 |  | 227 | 79.95 | 14.89 |  | 222 | 59.46 | 20.80 |  |
| no | 533 | 61.01 | 20.28 |  | 553 | 76.01 | 23.32 |  | 552 | 71.85 | 18.43 |  | 541 | 52.09 | 22.02 |  |
| Experiences with mental disorders due to professional activity |  |  |  | **0.0007** |  |  |  | 0.1854 |  |  |  | **0.0001** |  |  |  | 0.0933 |
| yes | 64 | 71.84 | 17.56 |  | 66 | 79.89 | 20.43 |  | 66 | 81.96 | 14.99 |  | 62 | 59.10 | 21.49 |  |
| no | 685 | 65.35 | 19.63 |  | 710 | 76.07 | 22.63 |  | 709 | 73.48 | 17.92 |  | 698 | **53.76** | **21.85** |  |
| Information about mental problems after AMI by physician |  |  |  | **0.021** |  |  |  | **0.0040** |  |  |  | **0.0002** |  |  |  | 0.1203 |
| yes | 236 | 67.03 | 16.72 |  | 244 | 79.51 | 21.16 |  | 244 | 77.72 | 16.34 |  | 237 | 56.05 | 21.61 |  |
| no | 515 | 62.71 | 20.66 |  | 536 | 74.82 | 22.85 |  | 535 | 72.58 | 18.31 |  | 526 | 53.31 | 21.99 |  |
| Information about mental problems after AMI during rehabilitation program |  |  |  | **0.0024** |  |  |  | **<.0001** |  |  |  | **0.0006** |  |  |  | **0.0011** |
| yes | 291 | 67.36 | 16.96 |  | 299 | 80.00 | 20.13 |  | 299 | 77.47 | 16.50 |  | 288 | 58.32 | 20.60 |  |
| no | 322 | 61.93 | 21.39 |  | 336 | 73.20 | 23.01 |  | 337 | 72.46 | 18.57 |  | 333 | 52.12 | 21.96 |  |
| Preferred to receive more information about mental problems after AMI |  |  |  | **0.0265** |  |  |  | **0.0003** |  |  |  | 0.6453 |  |  |  | **0.0196** |
| yes | 198 | 67.03 | 17.57 |  | 204 | 71.34 | 24.13 |  | 205 | 74.39 | 18.74 |  | 201 | 56.98 | 23.48 |  |
| no | 552 | 62.96 | 20.19 |  | 573 | 78.12 | 21.59 |  | 571 | 74.12 | 17.61 |  | 559 | 53.13 | 21.24 |  |
| PHQ |  |  |  | 0.9333 |  |  |  | **<0.0001** |  |  |  | **0.0097** |  |  |  | **0.0425** |
| No depression | 403 | 64.16 | 20.55 |  | 414 | 80.32 | 20.99 |  | 414 | 76.01 | 17.86 |  | 403 | 55.38 | 21.21 |  |
| Mild depression | 246 | 63.63 | 18.34 |  | 257 | 74.11 | 21.47 |  | 258 | 71.82 | 17.91 |  | 254 | 51.32 | 21.87 |  |
| Moderate depression | 74 | 65.40 | 18.39 |  | 79 | 68.75 | 25.71 |  | 78 | 74.66 | 17.02 |  | 75 | 57.57 | 22.38 |  |
| Moderately severe depression | 21 | 65.67 | 16.82 |  | 22 | 59.09 | 24.98 |  | 21 | 68.39 | 17.86 |  | 21 | 47.41 | 27.00 |  |
| Severe depression | 8 | 66.67 | 15.19 |  | 8 | 67.19 | 29.27 |  | 8 | 76.04 | 12.15 |  | 8 | 67.41 | 22.05 |  |

*Mann-Whitney U-Test or Kruskal-Wallis Test
